# HE4-based nomogram for predicting overall survival in patients with idiopathic pulmonary fibrosis: construction and validation

**DOI:** 10.1186/s40001-024-01829-0

**Published:** 2024-04-16

**Authors:** Mi Tian, Xiaohui Zhu, Lijun Ren, Xuan Zhou, Lina GU, Kaifang Meng, Yaqiong Tian, Hourong Cai, Xiaoqin Liu, Jingjing Ding

**Affiliations:** 1 Department of Respiratory and Critical Care Medicine, Nanjing Drum Tower Hospital, The Affiliated Hospital of Nanjing University Medical School, No. 321 Zhongshan Road, Nanjing, 210008 Jiangsu China; 2 Department of Respiratory, The Fourth Affiliated Hospital of Nanjing Medical University, 298 Nanpu Road, Nanjing, 211899 China; 3 Department of Pulmonary and Critical Care Medicine, Nanjing Drum Tower Hospital Clinical College of Nanjing University of Chinese Medicine, Nanjing, China; 4 Phase I Clinical Trials Unit, The Affiliated Drum Tower Hospital of Nanjing University Medical School, 359 Pu Zhu Middle Road, Nanjing, 210031 China; 5 Department of Nephrology, Affiliated Hospital of Nanjing University of Chinese Medicine, Jiangsu Province Hospital of Chinese Medicine, Nanjing, China; 6 Department of Respiratory and Critical Care Medicine, Nanjing Drum Tower Hospital Clinical College of Nanjing Medical University, No. 321 Zhongshan Road, Nanjing, 210008 Jiangsu People’s Republic of China

**Keywords:** IPF, HE4, Clinical prediction model, GAP index

## Abstract

Idiopathic pulmonary fibrosis (IPF) is a life-threatening interstitial lung disease. Identifying biomarkers for early diagnosis is of great clinical importance. The epididymis protein 4 (HE4) is important in the process of inflammation and fibrosis in the epididymis. Its prognostic value in IPF, however, has not been studied. The mRNA and protein levels of HE4 were used to determine the prognostic value in different patient cohorts. In this study, prognostic nomograms were generated based on the results of the cox regression analysis. We identified the HE4 protein level increased in IPF patients, but not the HE4 gene expression. The increased expression of HE4 correlated positively with a poor prognosis for patients with IPF. The HR and 95% CI were 2.62 (1.61–4.24) (*p* < 0.001) in the training set. We constructed a model based on the risk-score = 0.16222182 * HE4 + 0/0.37580659/1.05003609 (for GAP index 0–3/4–5/6–8) + (− 1.1183375). In both training and validation sets, high-risk patients had poor prognoses (HR: 3.49, 95%CI 2.10–5.80, *p* = 0.001) and higher likelihood of dying (HR: 6.00, 95%CI 2.04–17.67, *p* = 0.001). Analyses of calibration curves and decision curves suggest that the method is effective in predicting outcomes. Furthermore, a similar formulation was used in a protein-based model based on HE4 that also showed prognostic value when applied to IPF patients. Accordingly, HE4 is an independent poor prognosis factor, and it has the potential to predict IPF patient survival.

## Introduction

Idiopathic pulmonary fibrosis (IPF) is a chronic progressive and life-threatening interstitial lung disease characterized by common interstitial pneumonia of unknown cause [1, 2]. Men are more commonly affected than women. The clinical manifestations are progressive and aggravated dyspnea, decreased pulmonary function and even respiratory failure. The prognosis of IPF is depressing since an expected mean survival for patients upon diagnosis being 2–5 years only [3, 4]. There are no effective drugs to halt or reverse the natural process of IPF clinically. The existing anti-fibrosis drugs approved by Food and Drug Administration (FDA), pirfenidone and nintedanib [5], can only delay the decline of lung function. The only curative option is lung transplantation, which still faces many difficulties due to the scarcity of lung sources and high surgery costs [6]. A more accurate prognosis prediction is therefore needed to mitigate the risk and burden of IPF and to improve perceptions of best practices.

Researchers have attempted to quantify the severity of IPF at baseline and monitor changes in the condition over the past century. Multiple factors were considered as components of a prognostic scoring system, including age, gender, the results of pulmonary function tests (PFT), the severity of disease found on a high-resolution CT, the 6-min walking test, and the dyspnea levels [7]. In a study by King et al., a system for predicting the survival status of newly diagnosed IPF was developed based on the imaging physiological scores [8]. However, the clinical applicability of this scoring system was limited due to its complexity. In patients with IPF, the composite physiological index (CPI) has recently been found to be useful for predicting mortality [9, 10]. In the CPI, the percentage diffusing capacity of the lungs for carbon monoxide (DLco) and lung capacity was used as indicators, with the advantage that these indicators were simple and easy to use. However, a disadvantage of this method is that it cannot differentiate between discrete patients at higher and lower risk of adverse events. In 2012, Ley et al. [11] proposed a gender–age–physiology index (GAP) scoring model using four variables: gender (G), age (age, A), and two lung function indicators: forced vital capacity as a percentage of predicted value (FVC%) and DLco. Tran et al. [12] included 1620 patients with IPF and divided them into three stages according to GAP index score 0–3/4–5/6–8. It is suggested that GAP scoring model/stage can predict the death risk of IPF patients. GAP model has simple scoring and reliable differentiation, but its calibration is not satisfying. Therefore, it is of great clinical significance to find biomarkers for early diagnosis of IPF.

Human epididymis protein 4 (HE4) is a secreted glycoprotein belonging to the whey acid proteins (WAP) family, with a molecular weight of about 13 kDa. It is encoded by the WAP four disulfide core domain 2 (WFDC2) gene. HE4 expression was found in lung, kidney and salivary gland [13]. The functions of HE4 mainly include participating in inflammatory reaction and inhibiting protease activity. HE4 may be closely related to the occurrence and progression of some malignant tumors [14–17]. Serum HE4 and CA-125 are two biomarkers approved by FDA for ovarian cancer [18].

There have been studies on the involvement of HE4 in the occurrence and development of renal fibrosis, myocardial fibrosis and pulmonary cystic fibrosis (CF) [14, 19]. Serum HE4 protein expression increased in chronic kidney disease patients and renal fibrosis model mice. However, few literatures have reported the change of expression level and function of HE4 in IPF patients.

Our previous research shows elevated expression of serum HE4 in IPF patients, especially in those with acute exacerbation (AE–IPF). In addition, serum HE4 as well as GAP index was suggested valuable for predicting the prognosis of IPF patients [20]. In this article, we aim to comprehensively investigate the prognostic value of HE4 at both gene and protein levels. We also establish prognostic models to better guiding clinical practice.

## Methods

### Clinical samples and data collection

A total of 59 IPF patients and 29 age- and gender-matched normal people were included in our analysis. The detailed clinical information was gathered and listed in Table 1.

**Table 1:** Clinical characteristics of participants in GSE70866 and our own cohorts

	GSE70866–GPL14550		GSE70866–GPL17077	Clinical samples	
	NC (*n* = 20)	IPF (*n* = 112)	IPF (*n* = 64)	NC (*n* = 29)	IPF (*n* = 59)
Age	61.3 ± 8.3	68.0 ± 10.1	68.3 ± 8.5	64.3 ± 5.9	67.3 ± 8.1
Gender (*n*)					
Female	4	19	13	9	4
Male	16	93	51	20	55
GAP index (*n*)					
0–3		31	25		13
4–5		52	31		18
6–8		29	8		12

From November 2017 to April 2018, IPF patients diagnosed in Nanjing Drum Tower Hospital were enrolled in this study. IPF was diagnosed following the relative guidelines [21]. The survival data as well as age, gender, smoking history, and GAP index were obtained from medical records retrospectively. Overall survival (OS) time was calculated from the time of enrollment to the time of death or the last time of follow-up, March 1st, 2022. Our previous article [20] described the method used to measure HE4 and KL-6 protein levels in serum.

### Data collection from GEO datasets

GSE70866 data set analyzed 196 bronchoalveolar lavage fluid samples, including 20 normal controls and 112 IPF patients from GPL14550 platform, and 64 IPF patients from GPL17077 platform (Table 1) [22]. In GEO data sets (https://www.ncbi.nlm.gov/geo/), the Series Matrix File of GSE70866 was retrieved along with their corresponding clinical features [23]. The annotation files of GPL14550 and GPL17077 platforms were downloaded for gene annotation.

### Different expressed genes (DEGs) analysis

The R package “limma” was applied to identify DEGs between IPF patients and normal controls from GPL14550 platform. We set the criteria as |logFC|≥ 1 and adj.P.value < 0.05. The results were visualized via heatmap and volcano map. We also compared the expression of HE4 within clinical subgroups.

### Evaluation of HE4 gene’s prognostic efficacy

The prognostic value of HE4 gene in predicting overall survival (OS) of IPF patients was evaluated by Kaplan–Meier survival analysis and time-dependent receiver operating characteristic (timeROC) curve analysis. According to the median expression level of HE4, IPF patients were divided into high expression and low expression groups. The association was investigated between HE4 levels and OS. Hazard ratios (HRs) with 95% confidence intervals (95% CIs) and log-rank *p* values were calculated. The following R packages were used in the survival analysis procedure: survival (v3.2–10) and survminer (v0.4.9). The "ggplot2" package was utilized to display the results [24]. Moreover, timeROC curves were constructed with “timeROC” R package. The areas under the curve (AUCs) were calculated.

### Construction and validation of HE4-based prognostic signature

The IPF patient cohort from GPL14550 (GSE70866–GPL14550) was included as a training set, while the cohort from GPL17077 (GSE70866–GPL17077) was brought into a validation set. To establish a model for predicting OS in IPF, we conducted a univariate COX regression analysis that included age, gender, GAP score, and expression of HE4. We also tested KL-6 protein levels and smoking history of our own patients. We selected variables for multivariate COX regression analysis that had *p* values less than 0.1 based on the results. A multivariate analysis (*p* < 0.05) reveals that this variable is an independent factor affecting the prognosis of IPF patients. We developed a prognostic model based on multivariate COX regression analysis. The formula of prognosis model is: risk score = variable 1 * coefficient 1 + variable 2 * coefficient 2 + …… + variable n * coefficient n + constant. The concordance index (C-index) was used to assess the value of prognostic model. In addition, prognosis models were visualized through nomograms. The risk-score of each sample is calculated according to the prognosis model. Low- and high-risk subgroups were defined according to the median risk score for IPF patients. We compared the OS between the two subgroups using Kaplan–Meier survival analysis. The risk factor diagram is used to visualize the prognosis risk score of different samples in the model. Using the R package “rms”, the calibration curves were plotted to validate the veracity of the nomogram. An evaluation of the accuracy of a model is based on the calibration curve, while an evaluation of its clinical effectiveness is based on the decision curve analysis.

### Statistical analysis

As previously mentioned, analyses were conducted using R (version 4.1.2). The two-tailed Wilcoxon rank sum tests were applied for comparisons between two groups. If not otherwise specified, *p* values less than 0.05 were generally considered statistically significant.

## Results

### IPF patients show an elevated expression of HE4 protein but not HE4 gene

**Fig. 1:**
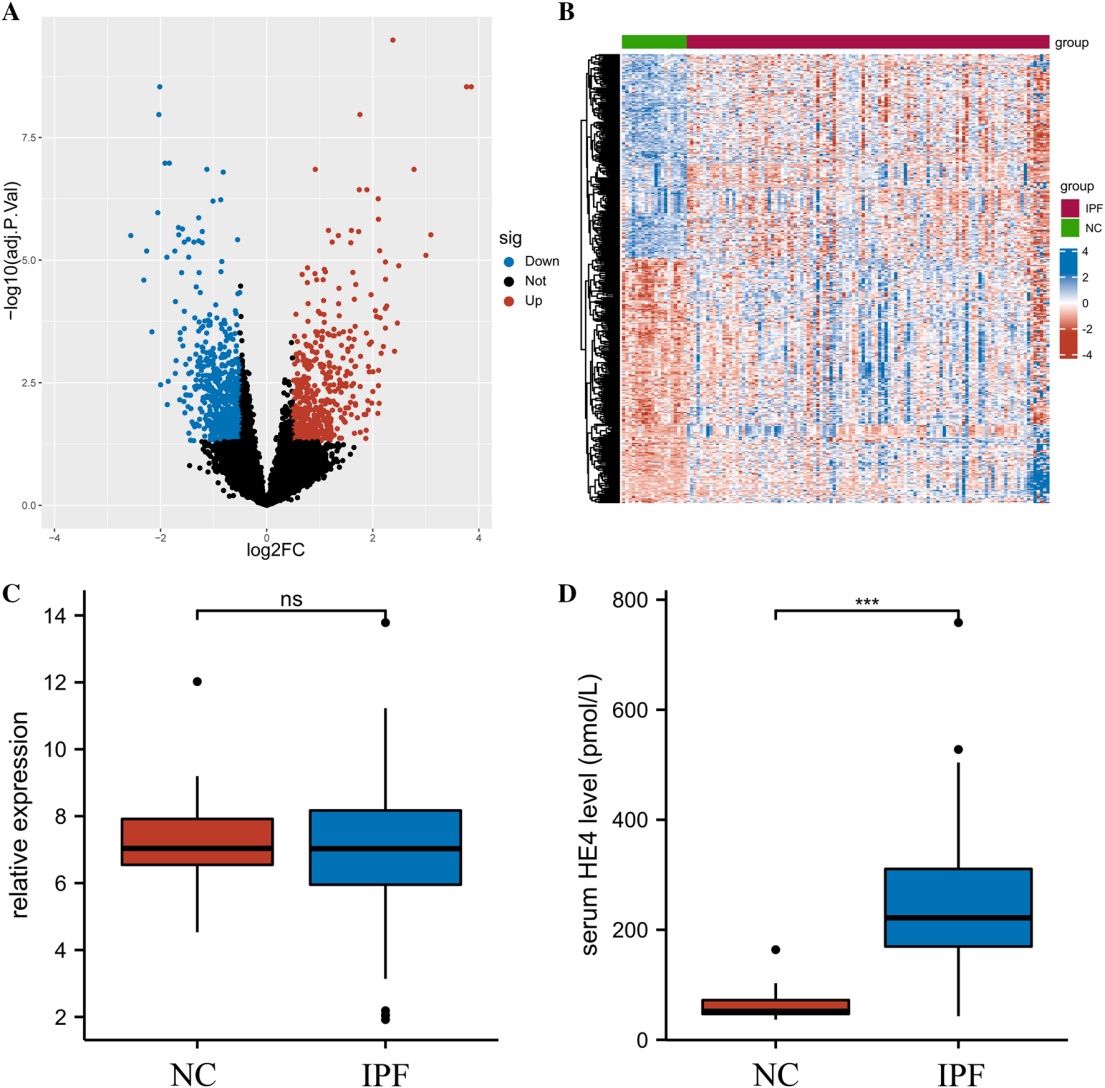
Expression of HE4 in IPF patients. **A** Volcano map displays DEGs between IPF patients and normal controls in the GSE70866–GPL14550 data set. **B** Heatmap of the expression of DEGs. **C** HE4 gene expression in IPF patients compared to the NC group. **D** Protein levels of HE4 in IPF patients compared to the NC group (*p* < 0.001). ***p < 0.001, ns: non-significant

We compared the gene expression between 20 normal controls and 112 IPF patients from GPL14550 platform in GSE70866 data set, and identified 379 DEGs of which 207 genes were upregulated and 172 were downregulated (Fig. 1A, B). The gene expression of HE4 did not differ in the two groups (Fig. 1C). However, the serum protein levels of HE4 increased significantly (*p* < 0.001, Fig. 1D).

**Fig. 2:**
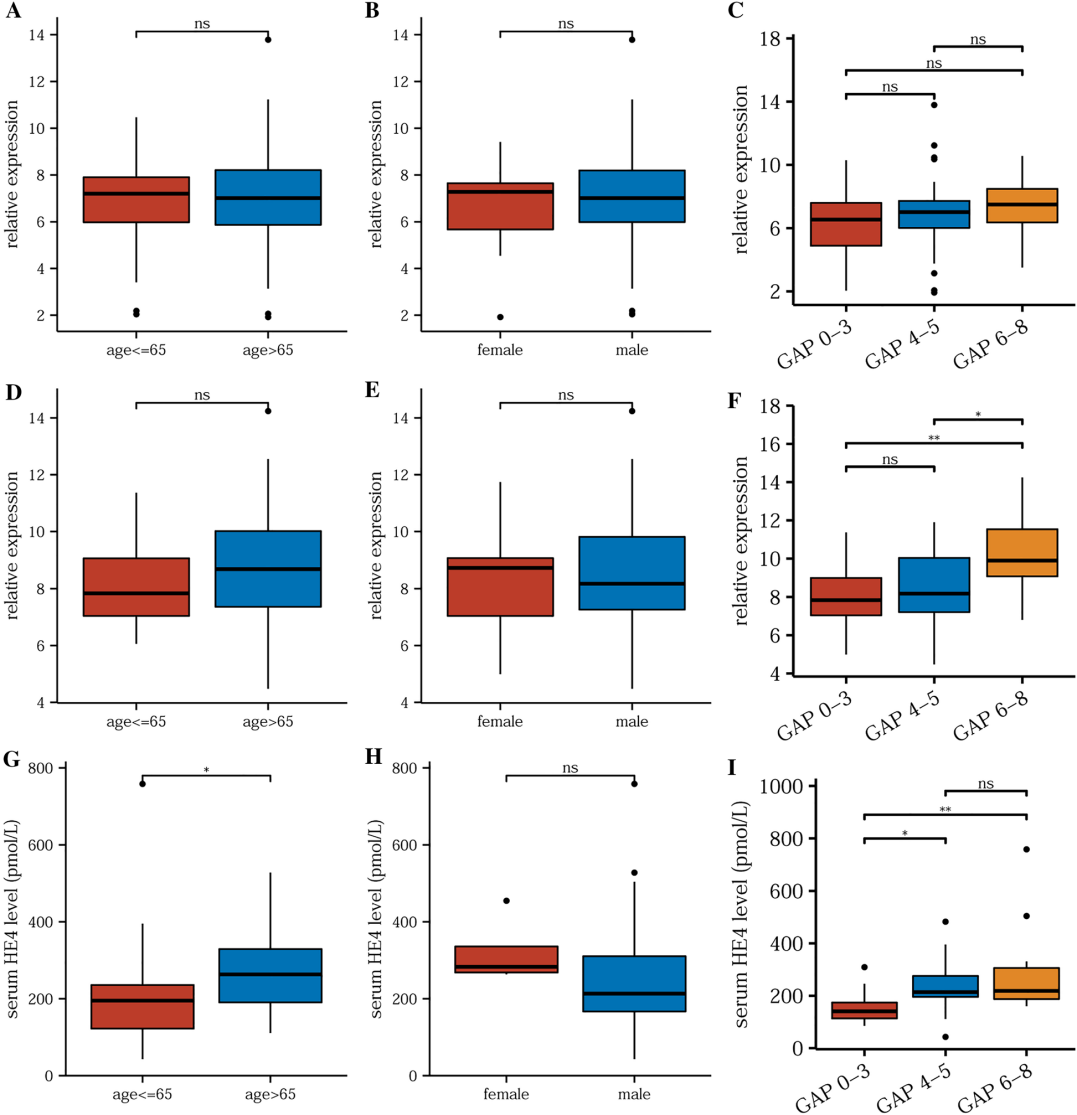
Association between HE4 expression and clinical characteristics of IPF patients. Data are shown for correlation between HE4 gene expression and **A** age, **B** gender, and **C** GAP index in training set; and **D**–**F** in validation set. The protein levels of HE4 in different subgroups of **G** age, **H** gender, and **I** GAP index. *p < 0.05, **p < 0.01, ns: non-significant

Next, we analyzed the correlation between HE4 expression and IPF patients’ clinical characteristics. There were no obvious differences between patients’ genders, ages, or GAP index levels in training cohorts (Fig. 2A–C). The HE4 gene levels were slightly higher in patients with a GAP index of 6–8 compared with 0–3 (*p* = 0.058). Similar results were found in validation cohort (Fig. 2D, E). An elevated expression of HE4 gene was significantly associated with high GAP index (GAP 4–5 vs. GAP 0–3: *p* = 0.019, GAP 6–8 vs. GAP 0–3: *p* = 0.009) (Fig. 2F). HE4 protein levels were also compared in IPF patients. HE4 did not show gender differences, but was higher in elderly patients (age > 65 vs. ≤ 65: *p* = 0.011) (Fig. 2G, H). HE4 protein level was also positively correlated with GAP index (GAP 6–8 vs. GAP 0–3: *p* = 0.007, GAP 6–8 vs. GAP 4–5: *p* = 0.043) (Fig. 2I).

### High expression of HE4 predicts poor prognosis

**Fig. 3:**
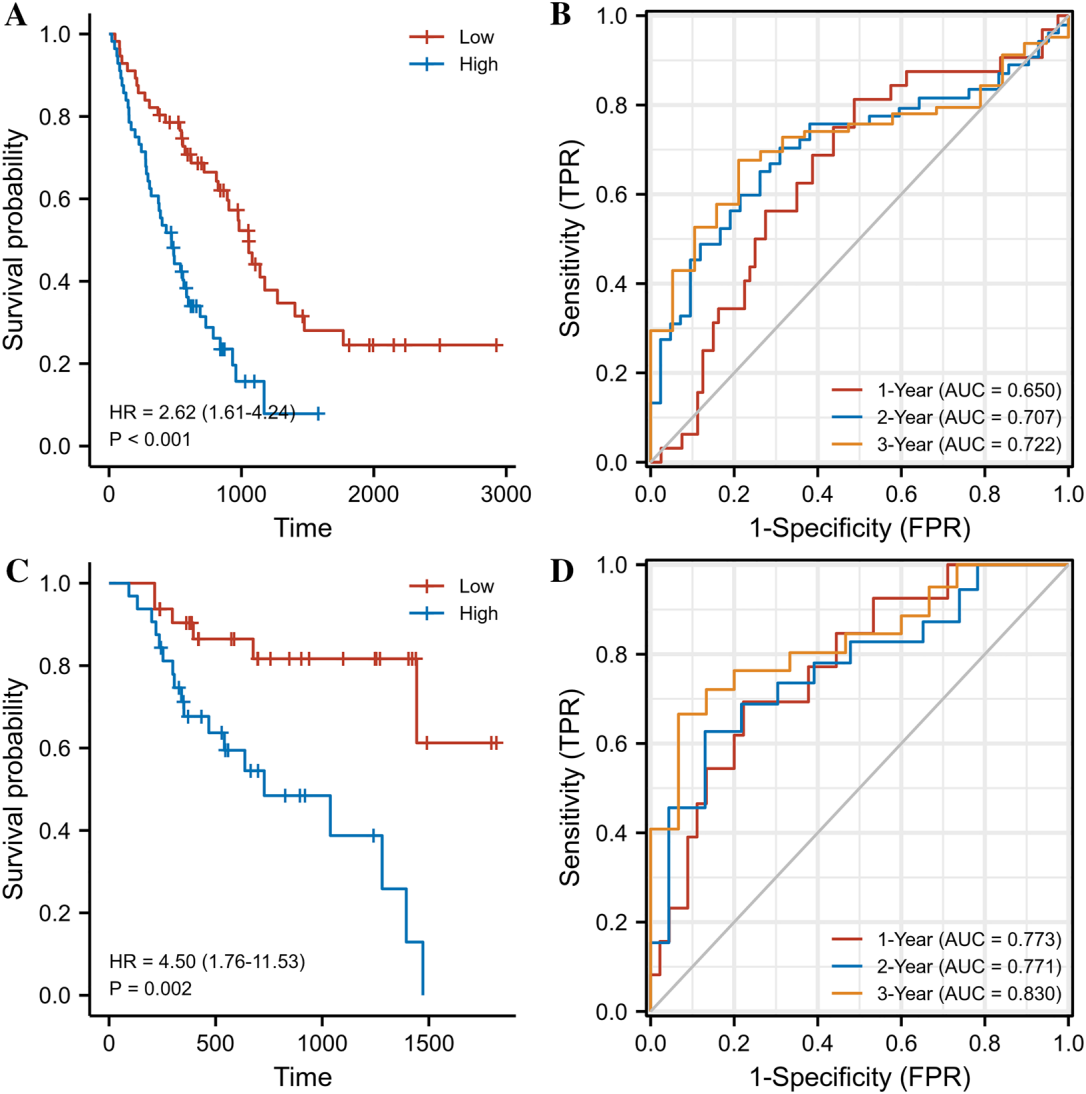
HE4 gene exhibits superior prognostic value in IPF in both training and validation sets. **A** Kaplan–Meier plotter in training set. **B** TimeROC curves in training set. **C** Kaplan–Meier plotter in validation set. **D** TimeROC curves in validation set

It was shown in our previous article that high levels of HE4 protein in IPF patients correlated with poor OS. We focus on the prognostic value of HE4 gene. Based on Kaplan–Meier plots (Fig. 3A, C), elevated expression of HE4 gene was significantly associated with poor OS in both training and validation cohorts. The HR and 95% CI were 2.62 (1.61–4.24) for training set and 4.50 (1.76–11.53) for validation set, and the p values were < 0.001 and 0.002, respectively.

The timeROC curves were drawn to further evaluate HE4’s values (Fig. 3B, D). The AUCs in predicting 1-, 2- and 3-year survival were 0.650, 0707, and 0.722 in training set, and 0.773, 0.771, and 0.830 in validation set. These results indicate that HE4 gene level possessed prognostic value in IPF.

### Construction and validation of a HE4 gene-based prognostic model

**Fig. 4:**
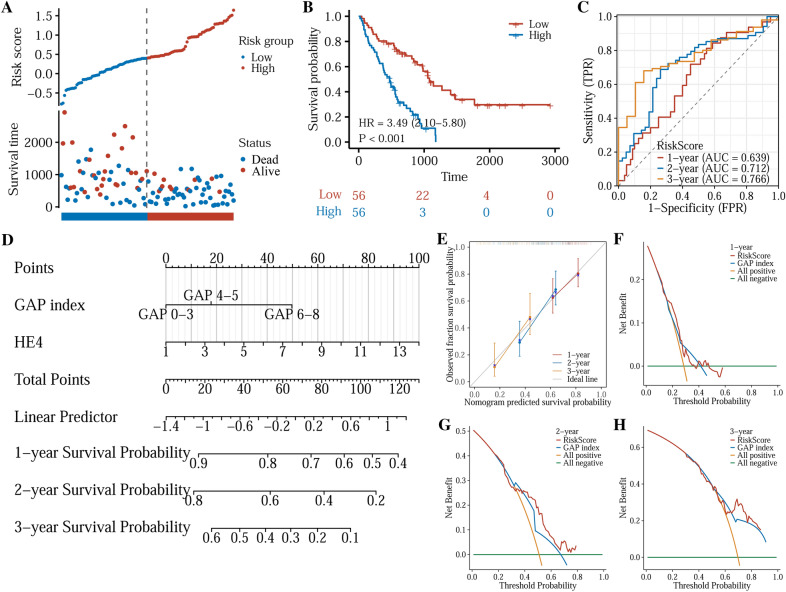
Construction of the risk model in the training cohort. **A** Distribution and survival status of patients based on the risk model. The left side of the dotted line: low-risk population. The right side: high-risk population. **B** Kaplan–Meier curves for the OS of patients in the low- and high-risk groups. **C** Time-dependent ROC curves of 1, 2, and 3 years. **D** Nomogram for prediction of overall survival rates in IPF patients based on the result of multivariate cox regression analysis. **E** Calibration curves of the nomogram prediction of 1-, 2-, and 3-year OS rates in IPF patients. **F** 1-Year DCA curve of the nomogram. **G** 2-Year DCA curve of the nomogram. **H** 3-Year DCA curve of the nomogram

We constructed a prognostic model using age, gender, GAP index, and HE4 gene expression to assess the utility of HE4 as an IPF prognostic factor. In a univariate and multivariate COX regression analyses, GAP index and HE4 gene level were found to be independent prognostic factors (Table 2). Based on HE4 gene and GAP index, a prognostic model was built that the results were visualized using a nomogram (Fig. 4D). The formula of the model is: risk-score = 0.16222182 * HE4 + 0/0.37580659/1.05003609 (for GAP index 0–3/4–5/6–8) + (− 1.1183375). The C-index of the model was 0.649. The risk-score was calculated for each patient and the median value was 0.4027. Patients were assigned to low- or high-risk groups, which is displayed in Fig. 4A. Kaplan–Meier plotter analysis showed IPF patients in high-risk group had a lower overall survival (OS) than those in low-risk group (HR: 3.49, 95%CI 2.10–5.80, *p* < 0.001) (Fig. 4B). Furthermore, the specificity and sensitivity of the model were also evaluated using time-dependent ROC analysis. In terms of 1-, 2-, and 3-year survival, the area under the ROC curve (AUC) were 0.639, 0.712, and 0.766, respectively (Fig. 4C). The calibration curve of the nomogram is shown in Fig. 4E, presenting good agreement between predicted and actual survival status. DCA was performed to measure the clinical effectiveness of the nomogram. It showed that the net benefits backed by the nomogram were slightly better than those by GAP index in predicting 2-year prognosis (Fig. 4F–H).

**Table 2:** Results of univariate and multivariate Cox regression analyses in the training set

Characteristics	Total (*n*)	Univariate analysis		Multivariate analysis	
		Hazard ratio (95% CI)	*p* value	Hazard ratio (95% CI)	*p* value
Age	112	0.986 (0.963–1.010)	0.255		
Gender	112				
Female	19				
Male	93	1.234 (0.665–2.292)	0.505		
GAP index	112				
0–3	31				
4–5	52	1.645 (0.908–2.982)	0.101	1.456 (0.797–2.660)	0.222
6–8	29	3.439 (1.798–6.578)	< 0.001	2.858 (1.480–5.520)	0.002
HE4	112	1.209 (1.080–1.353)	< 0.001	1.176 (1.040–1.330)	0.010

**Fig. 5:**
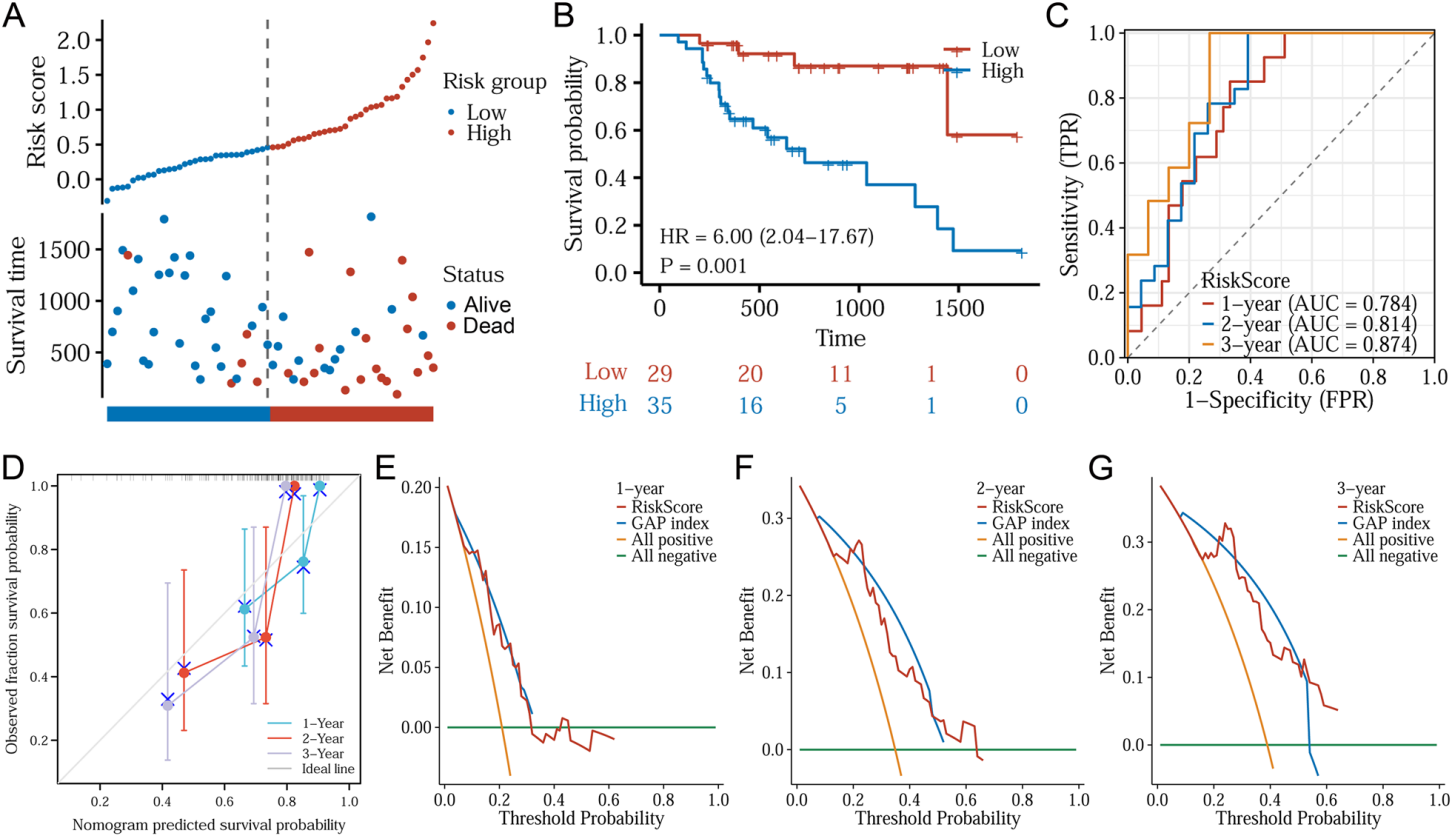
Assessment of the risk model in the validation cohort. **A** Risk scores were calculated for each patient using the model above, with a cutoff value of 0.4027 for low- and high-risk groups. The distribution and survival status of these patients were plotted. **B** Kaplan–Meier curves. **C** Time-dependent ROC curves of 1, 2, and 3 years. **D** Calibration curves. **E** DCA curve of 1 year. **F** DCA curve of 2 years. **G** DCA curve of 3 years

To evaluate its prognostic efficacy, the prognostic model was applied to the validation set. Patients in the validation cohort was calculated with a risk score and divided into low- and high-risk groups by cutoff value set as 0.4027. As a result, 29 patients were included in the low-risk group, and 35 in the high-risk group. It is indicated IPF patients with high risk-score have elevated rate of death (Fig. 5A). Kaplan–Meier plotter analysis further validated the results (HR: 6.00, 95%CI 2.–4–17.67, *p* = 0.001) (Fig. 5B). The AUCs of 1-, 2-, and 3-year survival were 0.784, 0.814, and 0.874 (Fig. 5C). Calibration (Fig. 5D) and DCA curves of 1, 2, and 3 years (Fig. 5E–G) were plotted which suggested similar efficacy to training set.

### Construction of a HE4 protein-based prognostic model

Since HE4 protein levels were correlated with IPF characteristics, we considered HE4 as a potential prognostic biomarker. We constructed a prognostic model which incorporated with age, gender, smoking history, GAP index, and the levels of HE4 and KL-6 proteins. KL-6 is a glycoprotein mainly secreted by type II alveolar epithelium and glandular cells. As part of the tissue repair process, it plays a key role in IPF pathophysiology. In a number of studies, it has been confirmed that an increase in KL-6 levels indicates a poor prognosis for patients with IPF. Among the 59 IPF patients, 16 patients were unable or refused to accept the pulmonary function test. Thus, we could not calculate the GAP index for these people. The 16 patients were therefore excluded for further analysis. Following the exclusion of patients with incomplete clinical information, 43 patients were finally included in the analysis.

**Fig. 6:**
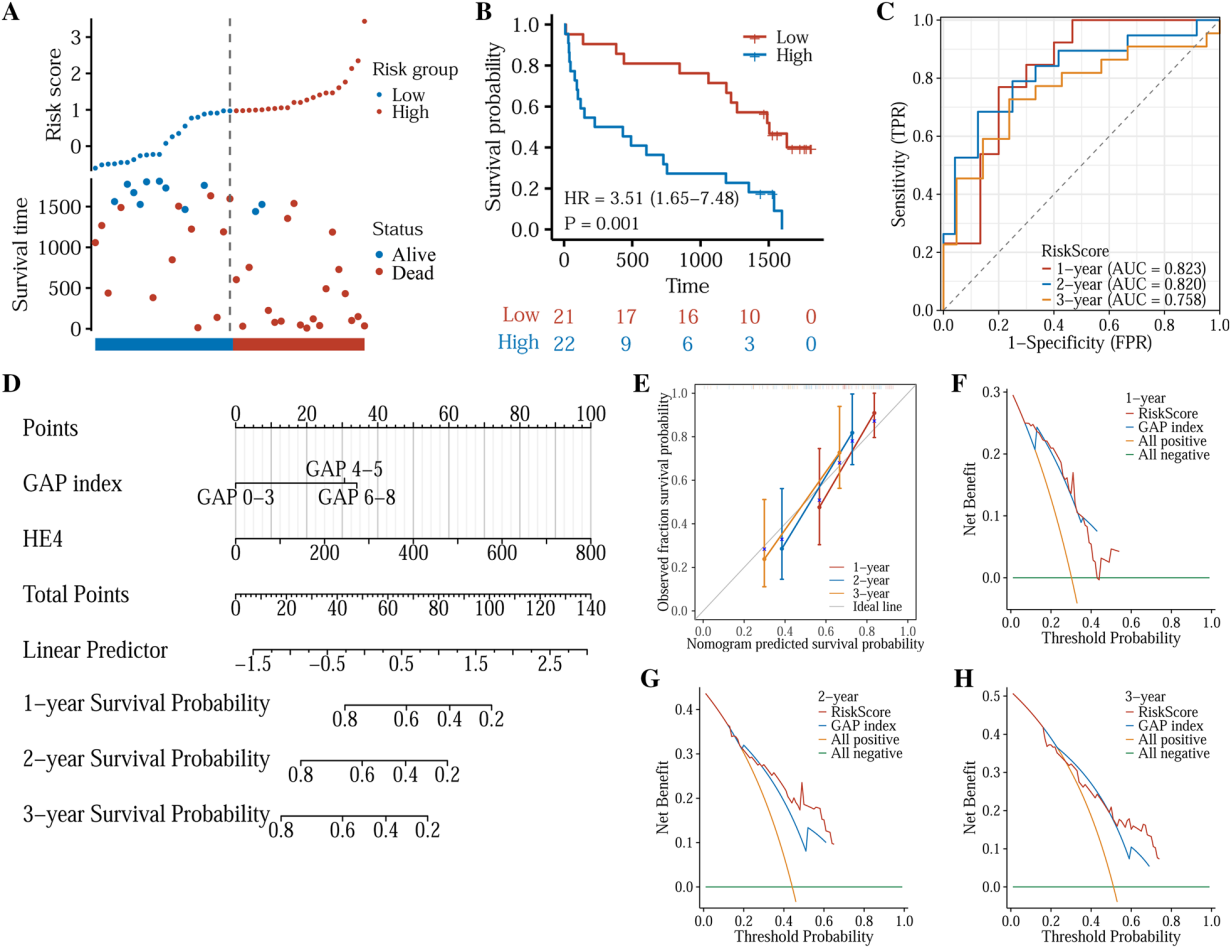
Construction of a new risk model in IPF patients. **A** Distribution and survival status of patients based on the model. **B** Kaplan–Meier curves for the OS of patients in the low- and high-risk groups. **C** Time-dependent ROC curves of 1, 2, and 3 years. **D** Nomogram for prediction of 4-year OS rates in IPF patients based on HE4 protein levels and GAP index. **E** Calibration curves of the nomogram. **F** 1-Year DCA curve of the nomogram. **G** 2-Year DCA curve of the nomogram. **H** 3-Year DCA curve of the nomogram

Table 3 shows results of univariate and multivariate COX regression analysis. Similar to the results above, HE4 protein level and GAP index were also independent prognostic factors who were subsequently utilized to draw a nomogram (Fig. 6D). The formula of the model is: risk-score = 0.00427293 * HE4 + 0/1.04647188/1.16579674 (for GAP index 0–3/4–5/6–8) + (− 0.9717294). The C-index of the model was 0.7. The distribution of patients in high- or low-risk group is displayed in Fig. 6A. A high risk-score was significantly correlated with poor prognosis (HR: 3.51, 95%CI 1.65–7.48, *p* = 0.001) (Fig. 6B). The AUCs of 1-, 2-, and 3-year survival were 0.823, 0.820, and 0.758 (Fig. 6C). Besides, calibration and decision curve analysis were performed, indicating a good prediction effect, especially in 2-year prognosis (Fig. 6E–H).

**Table 3:** Results of univariate and multivariate Cox regression analyses in clinical samples

Characteristics	Total (*N*)	Univariate analysis		Multivariate analysis	
		Hazard ratio (95% CI)	*p* value	Hazard ratio (95% CI)	*p* value
Gender	43				
Male	42	Reference			
Female	1	0.814 (0.110–6.014)	0.840		
Age	43	1.028 (0.983–1.075)	0.233		
Smoking	43				
Yes	22	Reference			
No	21	1.084 (0.537–2.187)	0.822		
HE4	43	1.005 (1.002–1.008)	< 0.001	1.004 (1.001–1.008)	0.010
KL-6	43	1.000 (1.000–1.000)	0.750		
GAP index	43				
GAP 0–3	13	Reference			
GAP 4–5	18	3.399 (1.312–8.806)	0.012	2.848 (1.084–7.481)	0.034
GAP 6–8	12	4.451 (1.590–12.456)	0.004	3.208 (1.075–9.579)	0.037

## Discussion

Currently, IPF cannot be cured. A prognosis model for IPF is essential, as it helps clinicians adapt treatment plans in time and so provide patients with clinical benefits. We developed two prognosis—prediction models based on GAP index separately from HE4 expression in IPF patients. In this study, the model was shown to be an independent factor predicting survival in patients with IPF. In the GSE70866 data set, HE4 gene expression does not change, but its protein levels rise in patients with IPF. It has been shown that high HE4 expression correlates with poor prognosis in further analyses. Overall, we believe that HE4 protein level could be a more reliable biomarker than its gene expression.

One possible concern is that there may be confounders influencing the prognostic model. Some risk factors have been identified, among which smoking has the strongest correlation with IPF; exposure to various dusts is also a risk factor, including stone, metal, wood, and organic dust. Gastroesophageal reflux promotes lung injury through trace aspiration, but the correlation is currently difficult to explain [25, 26]. Current research shows that the main environmental factors causing IPF include dust, fibers, smoke, and particles [27]. Studies have found that the incidence of IPF is significantly increased in populations exposed to inorganic dust and animal dust, chemical smoke (including wood chips and smoke), copper, lead, and steel metal dust (excluding bird feces) and other pollutants. Our patients have been excluded from other known causes of interstitial lung disease such as family or occupational environmental exposure, connective tissue disease, and drug toxicity according to the diagnostic criteria for IPF; patients enrolled in our experiment have no secondary factors caused by clear occupational environmental exposure; however, a significant number of IPF patients have no history of environmental exposure, suggesting that the mechanism of environmental exposure on the occurrence and development of IPF remains to be further elucidated.

HE4 has been used as an important protein in many clinical prediction models. Investigations were conducted into HE4's role in pulmonary diseases. Patients with lung cancer had significantly higher serum HE4 levels than those with benign lung disease and healthy controls, according to the results. The results showed that the serum HE4 level of patients with advanced disease was significantly higher than that of healthy control group [28, 29]. In addition, 34 ILD patients with progressive fibrosis and 40 healthy volunteers were retrospectively studied to determine serum levels of HE4. Results showed that serum HE4 levels were related to chest high-resolution computed tomography honeycomb levels. Researchers found that higher levels of HE4 were associated with a higher mortality risk. It has been proven that serum HE4 levels can be used to diagnose and prognosticate the prognosis of ILD patients with progressive fibrosis [30]. An increase in mortality risk was associated with serum HE4 levels and the GAP index.

Inflammatory reaction and hypoxia are considered as potential risk factors for IPF patients. Studies have established a robust method to predict clinical outcomes in IPF patients based on inflammation and hypoxia related gene characteristics. Five genes, including HE4, were identified as inflammation hypoxia-related genes, which can accurately predict the clinical outcome of IPF patients [31]. Hence, we believe that HE4 may play a key role in IPF. Several studies have demonstrated that HE4 expression is upregulated in fibrosis-associated fibroblasts (FAF). An elevated HE4 expression is associated with promoted macrophage proliferation [32]. Elevated levels of HE4 result in increased M2 macrophages and decreased CD8 + T cell infiltration, forming an immunosuppressive microenvironment [33]. In addition, HE4 may stimulate inflammation through NF-κB and MAPK signaling pathways [34, 35]. HE4 can inhibit multiple proteinases because of the disulfide linkages in its domains. In FAF, HE4 specifically inhibits the activity of MMP2 and MMP9 serine proteases, as well as their ability to degrade type I collagen. Furthermore, HE4 induces PD-L1 expression through a post-transcriptional mechanism, which mediates the transition of lung fibroblasts into myofibroblasts via Smad3 and β-catenin signaling pathways in IPF [36].

Despite the fact that this study provides new insights into the relationship between HE4 expression and IPF prognosis, some limitations must be considered. First of all, we cannot draw accurate conclusions from the sequencing data we have used in this study because they are mainly from online databases. Second, only GEO data sets are used, which may cause selection bias. Further studies in clinical samples are needed to determine what role the HE4 plays in IPF. Finally, there is still a need to investigate the mechanism by which HE4 regulates the progression of IPF patients.

In conclusion, HE4 is an independent poor prognosis factor, and has the potential to predict the survival outcome of IPF patients.

## Data Availability

The GSE70866 data set is available from the GEO website (https://www.ncbi.nlm.nih.gov/geo/query/acc.cgi?acc=GSE70866). The annotation files of GPL14550 and GPL17077 platforms were downloaded from https://www.ncbi.nlm.nih.gov/geo/query/acc.cgi?acc=GPL14550 and https://www.ncbi.nlm.nih.gov/geo/query/acc.cgi?acc=GPL17077.
